# Using Functional Near-Infrared Spectroscopy to Measure Effects of Delta 9-Tetrahydrocannabinol on Prefrontal Activity and Working Memory in Cannabis Users

**DOI:** 10.3389/fnhum.2017.00488

**Published:** 2017-10-10

**Authors:** Hasan O. Keles, Milena Radoman, Gladys N. Pachas, A. Eden Evins, Jodi M. Gilman

**Affiliations:** ^1^Center for Addiction Medicine, Department of Psychiatry, Massachusetts General Hospital, Boston, MA, United States; ^2^Department of Psychiatry, Harvard Medical School, Harvard University, Boston, MA, United States

**Keywords:** functional near-infrared spectroscopy, cannabis, marijuana, *n*-back, working memory, prefrontal cortex, THC, dronabinol

## Abstract

Intoxication from cannabis impairs cognitive performance, in part due to the effects of Δ9-tetrahydrocannabinol (THC, the primary psychoactive compound in cannabis) on prefrontal cortex (PFC) function. However, a relationship between impairment in cognitive functioning with THC administration and THC-induced change in hemodynamic response has not been demonstrated. We explored the feasibility of using functional near-infrared spectroscopy (fNIRS) to examine the functional changes of the human PFC associated with cannabis intoxication and cognitive impairment. Eighteen adult regular cannabis users (final sample, *n* = 13) performed a working memory task (*n*-back) during fNIRS recordings, before and after receiving a single dose of oral synthetic THC (dronabinol; 20–50 mg). Functional data were collected using a continuous-wave NIRS device, in which 8 Sources and 7 detectors were placed on the forehead, resulting in 20 channels covering PFC regions. Physiological changes and subjective intoxication measures were collected. We found a significant increase in the oxygenated hemoglobin (HbO) concentration after THC administration in several channels on the PFC during both the high working memory load (2-back) and the low working memory load (0-back) condition. The increased HbO response was accompanied by a trend toward an increased number of omission errors after THC administration. The current study suggests that cannabis intoxication is associated with increases in hemodynamic blood flow to the PFC, and that this increase can be detected with fNIRS.

## Introduction

Cannabis is the most widely used ‘illicit’ drug in the United States, with nearly 20 million people reporting past month use (Substance Abuse and Mental Health Services Administration, 2011). Acute, detrimental effects of Δ9-tetrahydrocannabinol (THC), the primary psychoactive compound in cannabis, on performance of cognitive and psychomotor functioning are well documented. In double-blind, placebo-controlled studies, oral administration of 40–300 μg/kg THC caused acute, dose-dependent impairment in performance on memory, divided and sustained attention, reaction time, visual tracking and motor function tasks (Hall and Solowij, 1998; Leweke et al., 1998; Ameri, 1999; Hampson and Deadwyler, 1999; Curran et al., 2002; Lichtman et al., 2002; D’Souza et al., 2004; Ramaekers et al., 2004). Many of these tasks rely on the prefrontal cortex (PFC), which is heavily involved in processes such as executive control, working memory, novelty processing, attention and awareness, and integration of multimodal sensory information (Knight and Stuss, 2002). Cannabinoid 1 (CB1) receptors are located throughout the PFC, as well as in the striatum, amygdala, and cerebellum (Gardner, 2005). Therefore, studying the effects of THC on blood flow in the PFC during cognitive tasks testing any of the above processes is essential to increasing our understanding of how cannabis impacts cognitive performance.

Most brain imaging studies of acute cannabis exposure have demonstrated global cortical activity increases during administration of smoked cannabis or infused THC (Mathew et al., 1997, 1992). Mathew et al. (1992, 1997), using Positron Emission Tomography (PET), have consistently reported increased bilateral frontal lobe activation following administration of smoked cannabis or infused THC. In these studies, ratings of intoxication correlated with right hemispheric cerebral blood flow (CBF) increases (Mathew et al., 1997). Increases in blood flow to the PFC after a THC infusion are particularly pronounced in chronic marijuana users (Volkow et al., 1996).

In the current study, we examined changes in the PFC associated with THC administration during a working memory task using functional near-infrared spectroscopy (fNIRS) in 13 regular cannabis users. Functional NIRS is a portable, non-invasive, and inexpensive imaging modality that detects brain activity through local hemodynamics, similar to the BOLD activation measured by fMRI (Strangman et al., 2002; Keles et al., 2016). fNIRS has several advantages over other imaging modalities such as PET and functional magnetic resonance imaging (fMRI), in that fNIRS is economical, generally well-tolerated (e.g., participants can remain comfortably seated), and does not expose participants to radiation like in PET. Its main disadvantage is that near-infrared light pulsed into the forehead can only refracted from the tissues of the cortex up to depths of 3 cm, so that this method cannot be used to investigate subcortical regions. Because of this limitation, fNIRS is not as widely used as fMRI or PET, though fNIRS has been used to study acute effects of both medication [methylphenidate in children with ADHD; (Monden et al., 2012)] and addictive substances [heroin and methadone compared to saline in opiate-dependent and healthy subjects; (Stohler et al., 1999)]. To our knowledge, the current study is the first to use fNIRS to investigate functional changes in the human brain associated with THC administration. The goal of this preliminary study was to use fNIRS to detect potential changes in the human PFC associated with THC administration during a working memory task. Based on PET studies of cannabis intoxication (e.g., Mathew and Wilson, 1993; Volkow et al., 1996; Mathew et al., 1997, 1999) we hypothesized that, using fNIRS, we would observe significantly increased hemodynamic response during a working memory task after THC administration.

## Materials and Methods

Study procedures were approved by the Partners Human Subjects Committee (IRB approval #2015P001516). All participants completed an informed consent process and signed a written informed consent form prior to initiation of study procedures. Participants were compensated $100, which was mailed to their residence in the form of a check after completion of the study.

### Participants

Eighteen regular (at least weekly) cannabis-using adults, ages 18–55, were recruited through public advertising. Participants were asked to refrain from using any intoxicating substance, including marijuana, on the day of the study. We performed a qualitative five-panel urine drug screen (Medimpex United Inc.) and excluded those who tested positive for opiates, cocaine, amphetamines, methamphetamines, or tested negative for cannabis metabolites. We also performed a quantitative urine analysis of 11-nor-9-carboxy-tetrahydrocannabinol (THC-COOH) to determine the amount of THC metabolites in their system. Exclusion criteria included: any serious unstable medical illness (e.g., unstable angina, significant cardiovascular event in the prior 6 months, clinically significant cardiac conduction disorder, uncontrolled hypertension, tachycardia, renal failure), any serious mental disorder whether stable or not (e.g., schizophrenia, bipolar disorder), and those with allergy to sesame oil (because THC for oral consumption is suspended in sesame oil).

### Dronabinol Administration

Eligible participants were given a single dose of up to 50 mg of dronabinol, an FDA-approved synthetic THC ingredient in MARINOL^®^ Capsules (Solvay Pharmaceuticals, 2004). The study physicians (GP and AEE) determined the dronabinol dose based on the degree of expected tolerance, given participant’s average dose, frequency, and type of cannabis use, self-report of degree of intoxication (high) experienced with each use, history of any adverse effects experienced when using cannabis and the dose at which those adverse effects were experienced, and baseline characteristics such as participant’s sex, height, weight, BMI and blood pressure. Please see **Table 1** for dosing information for each participant.

**Table 1:** Demographics and clinical characteristics of participants.

Participant	Age range	Height	Weight	BMI	MJ Use	MJ Use	MJ Use	Quant. THC^1^	Dronabinol
ID	(years)	(m)	(kg)		(days/wk)	(times/day)	(joints/wk)	(ng/ml)	Dose (mg)
1	40–45	1.70	72.7	25.1	7	2	7.0	–	40
2	20–25	1.65	72.7	26.6	2	1	3.0	–	50
3	20–25	1.80	75.0	22.3	7	3	21.0	–	50
4	20–25	1.85	86.4	25.1	3	4	3.6	>500	40
5	26–30	1.88	70.5	19.9	7	3	16.8	>500	50
6	20–25	1.83	84.1	25.1	3	1	1.5	70	35
7	20–25	1.70	59.1	20.4	4	2	6.4	65	45
8	20–25	1.68	68.2	24.2	5	1	3.5	27	35
9	20–25	1.78	70.5	22.2	4	1	2.4	96	20
10	18–20	1.83	67.3	20.1	6	1	1.2	>500	35
11	20–25	1.80	85.0	26.1	4	1	2.0	54	35
12	20–25	1.63	57.3	21.6	6	1	3.0	35	30
13	26–30	1.78	75.0	23.7	7	1	1.7	35	40

*^1^Quantitative THC urine analysis (not available for the first three participants).*

### Assessments

Participants completed a baseline self-report cannabis use questionnaire (developed in-house, 11-items) querying age of onset of regular cannabis use, recency of use, average pattern and quantity of cannabis use, as well as the circumstances of any regular or prior negative psychological and physical experiences when using cannabis. Participants then underwent a detailed medical and drug use history with a study physician. The Drug Effects Questionnaire (DEQ) (Morean et al., 2013), a 100 mm visual analog scale, was administered pre-dose and every 20–25 min post-dose to assess the extent to which participants (1) felt any dronabinol effect(s), and (2) felt high. Heart rate and blood pressure measurements were also collected at baseline and at 25-min intervals after dronabinol administration.

### Experimental Design and Working Memory Task

**FIGURE 1:**
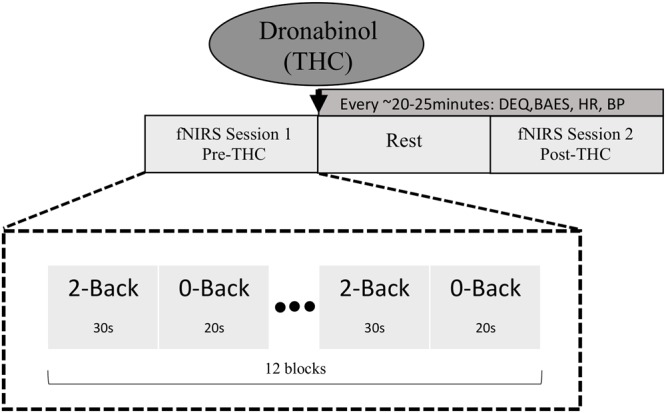
Schematic diagram of study and task design. fNIRS data were collected at two time points; pre-THC (fNIRS Session 1) and post-THC (fNIRS Session 2) administration (mean time = ∼1.5 h post-dronabinol dose), during which participants performed the *n*-back task. Two conditions (2-Back; high WM load and 0-Back; low WM load) were alternated in a blocked fashion.

Our experimental procedure is summarized in **Figure 1**. Participants underwent two fNIRS sessions; one before dronabinol administration (“pre-THC”), and the other at approximately 2 h after dronabinol administration (“post-THC”), which is the median peak of pharmacokinetic effects of dronabinol (Solvay Pharmaceuticals, 2004). During each session, participants completed a letter *n*-back working memory (WM) task, consisting of two conditions. For the 0-back condition (low WM load), participants were instructed to press a response button whenever a letter “X” appeared on a 15-inch computer screen. For the 2-back condition (high WM load), they were instructed to press the button whenever the presented letter was identical to the letter presented two trials prior. Letters were presented in pseudo-randomized order with a presentation time of 500 ms and an inter-stimulus-interval (ISI) of 2000 ms. The two conditions were presented in a blockwise fashion. Each 0-Back block was 20 s long, while each 2-Back block was 30 s long. Each of the two task-conditions was conducted six times, resulting in six 50 s task segments for both the 0-back and the 2-back conditions. There were 20 target trials (true positives) in each condition across all blocks; in the 0-Back condition, there were 20 presentations of “X,” and in the 2-back condition, there were 20 instances in which the presented letter was identical to the letter presented two trials prior. Additionally, a 10 s baseline period preceded the first task segment. All participants practiced the task for 1 min and were given feedback on their performance. Stimuli were generated, and responses were collected using PsychoPy (Psychophysics Software in Python).

### Acquisition and Analysis of fNIRS Imaging Data

A CW-NIRS (NIRSport 8-8, NIRx, Medical Technologies LLC, New York) device was used to simultaneously acquire dual-wavelength (760 and 850 nm) near-infrared light in order to measure relative concentration changes in oxy- and deoxy- hemoglobin (HbO and HbR) (Maki et al., 1995; Yamashita, 1996) based on the modified Beer-Lambert law (Cope et al., 1988). The sampling frequency was 7.81 Hz. NIRStar software by NIRx was used to verify the signal quality before each recording. NIRS data event markers were displayed, recorded and stored on the recording PC.

**FIGURE 2:**
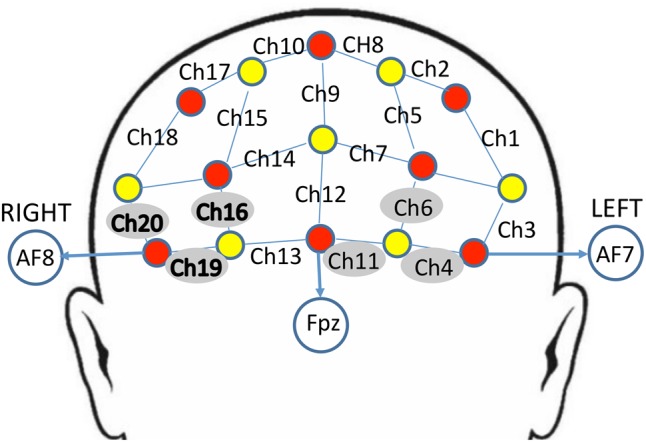
Schematic arrangement of the near-infrared spectroscopy (NIRS) probe array (front view). The NIRS probe was comprised of eight sources (red) and seven detectors (yellow) placed on the PFC of each participant. The mid-column of the probe was placed over Fpz, with the lowest probes located along the AF7-Fp1-Fpz-Fp2-AF8 line, in accordance with the International 10–20 Placement System. The distance between pairs of source and detector probes was set at 3 cm. The midpoint of the source-detector distance was defined as channel (Ch) location, labeled numerically (1–20) in the above schematic. Channels surrounded by gray circles indicate those that showed trend-level effects of dronabinol in the current study; bolded channels on the right (Ch 16, Ch 19, and Ch 20) showed a significant effect of dronabinol after multiple comparisons corrections.

The NIRS probe comprised eight sources and seven detectors placed over the PFC region of each participant (see **Figure 2** for a schematic). The mid-column of the probe was placed over Fpz, with the lowest probes located along the AF7-Fp1-Fpz-Fp2-AF8 line, in accordance with the International 10–20 Placement System (Trans Cranial Technologies, 2012). The center of the cap was placed over the vertex (Cz) of each participant, at a point equidistant from both nasion (Nz) ad inion (Iz) and equidistant from the left and right preauricular (LPA and RPA) points (Jurcak et al., 2007). The distance between pairs of source and detector probes was set at 3 cm. The midpoint of the source-detector distance was defined as channel (Ch) location. To minimize motion artifacts in the signal, the participants were instructed to remain as still as possible.

Data analysis was conducted using the open source software Homer2 (MGH-Martinos Center for Biomedical Imaging, Boston, MA, United States), implemented in Matlab (Mathworks, Natick, MA, United States). Motion artifacts were detected as signal changes larger than 10% of the standard deviation of the signal within a time-period of 0.5 s. These artifacts were detected and removed using a channel-based cubic spline interpolation in Homer2 (Huppert et al., 2009). fNIRS signals were preprocessed with a high-pass filter using cut-off frequencies of 0.01 Hz to remove baseline drift, and a 0.5 Hz low-pass filter to reduce impact of heartbeat pulsations, respiration, blood pressure, and skin blood flow on the signal. The modified Beer-Lambert law was applied to calculate hemoglobin concentration changes. Power Spectral Density (PSD) was also computed from all channels. The data were block averaged to obtain an average response to the *n*-back task for each of the 20 channels in each participant pre and post-THC. The high WM load (2-back) block averaged data consisted of signal from the time window from 0 to 30 s, and the low WM load (0-back) consisted of signal from 30 to 50 s. We chose the amplitude of the HbO concentration change as the primary metric, as HbO concentration has been observed to provide greater SNR than HbR (Strangman et al., 2002).

### Statistical Analysis

Statistical analyses were performed using GraphPad Prism version 7.0 (GraphPad Software, La Jolla, CA, United States). For all statistical analyses, we used the mean HbO concentration changes during the fNIRS recordings as our dependent variables, which provided one value per channel per participant per condition for both pre and post-THC. These values were analyzed using two-way repeated measures ANOVAs with treatment (pre and post-THC) and memory-load (0-Back and 2-Back) as within-subject factors in each channel (Ch1-20). We corrected for multiple comparisons using the Benjamini and Hochberg False Discovery Rate procedure (Benjamini and Hochberg, 1995). Behavioral data (*n*-back error rates and reaction time (RT)) were also analyzed using two-way repeated measures ANOVAs with treatment and memory-load as factors. These data were further analyzed using non-parametric testing (Wilcoxon matched-pairs signed rank test), because of the small sample size and the lack of normal distribution among participants.

## Results

Eighteen participants completed consent, met eligibility criteria and were enrolled. Five participants were excluded from the final analysis; three received a very low dose of dronabinol (5 mg) because their baseline blood pressure was >140/90; two subjects completed study procedures but their data were excluded from the analysis due to heavy motion artifacts in the fNIRS signal. The final cohort consisted of 13 cannabis users (10 males, 3 females, mean age 24 ± 6.75 years) who completed the fNIRS sessions. Please see **Table 1** for participants’ baseline demographic and clinical information. Quantitative urine analysis of THC-COOH was implemented after the first three participants, and therefore, quantitative measures of circulating THC are only available for 10 participants.

### Subjective Intoxication and Physiological Response to Dronabinol

**FIGURE 3:**
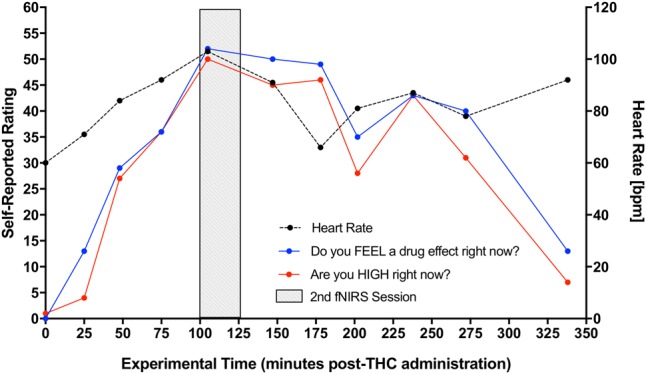
Increase in participants’ self-reported rating of intoxication and measured heart rate post-dronabinol administration. Every 25 min participants answered two questions: (1) “Do you FEEL a drug effect right now?” and (2) “Are you HIGH right now?” on a 0–100 mm visual analog scale, 0 mm being “Not at all” and 100 mm being “Extremely.” Subjects’ heart rate was also collected at each 25-min interval. The graph depicts averaged participants’ ratings and heart rate measurements. Second fNIRS session was completed at approximately 100–125 min post-dronabinol administration (shaded block).

Participant’s DEQ intoxication ratings of (1) feeling a drug effect and (2) feeling high increased significantly from pre-THC to post-THC, with mean peak ratings of 66.23 ± 27.73 mm (*t* = 6.15, *p* < 0.001) and 63.31 ± 24.62 mm (*t* = 6.13, *p* < 0.001), respectively. They also experienced an expected increase in heart rate from baseline to peak (*t* = 5.19, *p* < 0.001); mean increase at peak “high” was 33.77 ± 6.75 bpm (**Figure 3**). There were no significant changes in either systolic or diastolic blood pressure from pre to post-THC administration.

### Behavioral Results

**FIGURE 4:**
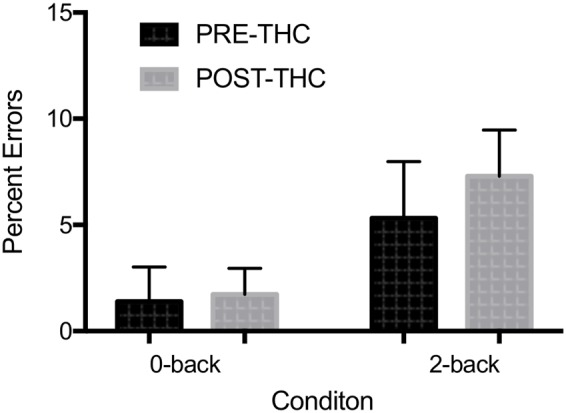
Mean percent errors during *n*-back working memory (WM) task. A robust main effect of memory-load was found. No significant interactions between memory-load type and drug treatment condition were found. Error bars represent standard deviations.

Error Rate: Due to a technical error, post-THC behavioral *n*-back data for one of the participants was lost (remaining sample *n* = 12). Out of 20 total target trials on the 0-back task, participants made an average of 0.67 ± 1.2 errors pre-THC and 0.83 ± 2.07 errors post-THC. Out of 20 trials on the 2-back task, participants made an average of 3.83 ± 3.0 errors pre-THC and 5.25 ± 5.43 errors post-THC. Percent error on the task indicated a main effect of memory-load (*F* = 7.89, *p* = 0.01), showing that participants were less accurate during the high than low WM load (**Figure 4**). Though participants generally made more errors after THC, overall task performance was not significantly different between pre and post-THC (*F* = 1.06, *p* = 0.31). Wilcoxon matched-pairs signed rank test, also failed to show a significant difference in performance after THC administration (*p* = 0.40).

Reaction Time: RTs were generally faster during the 0-back than the 2-back task, though the interaction between RT and memory-load did not reach significance (*F* = 3.64, *p* = 0.07). There was also no interaction between reaction time and drug treatment condition (*F* = 0.08, *p* = 0.78), though Wilcoxon matched-pairs signed rank test indicated a trend toward slower RTs after THC administration (*p* = 0.06).

### fNIRS Neuroimaging Results

**FIGURE 5:**
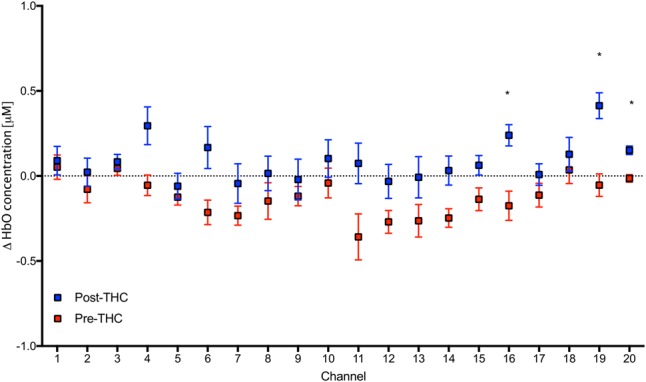
Mean values in HbO concentrations in each channel during 2-Back working memory task pre and post-THC administration. Channels with significant increase in HbO post-THC vs. pre-THC (*p* < 0.05) are marked with an asterisk. Error bars represent standard error of the mean.

**FIGURE 6:**
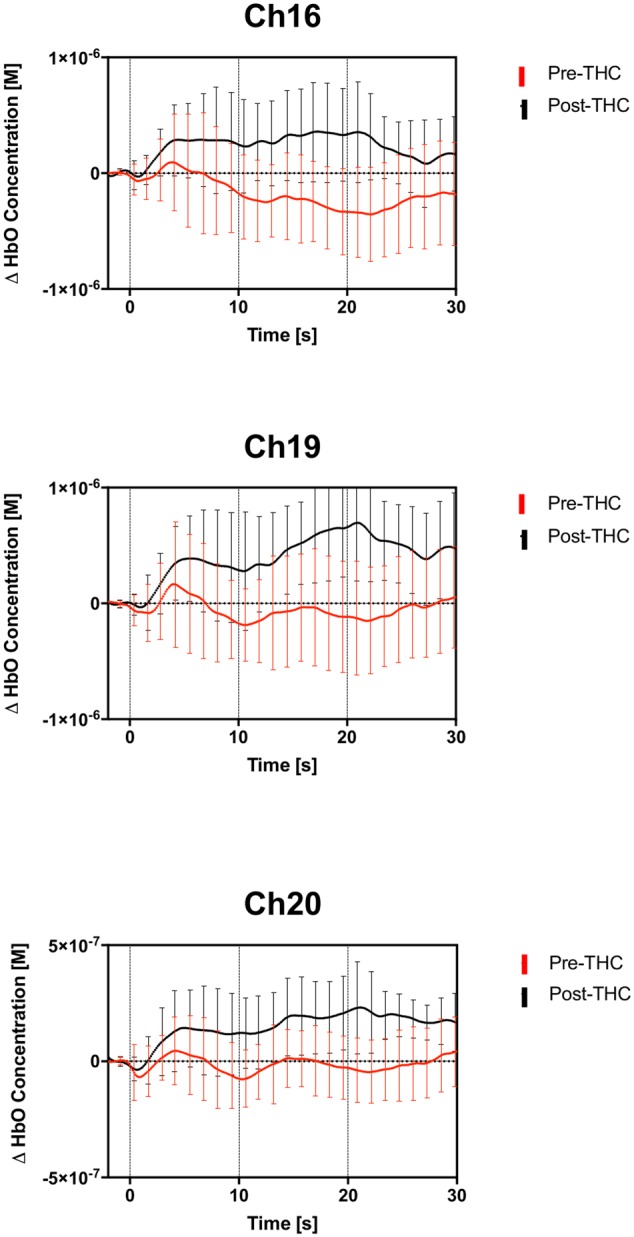
Group averaged (*n* = 13) time course of HbO concentration during the 2-back working memory blocks, from 0 to 30 s. Average time course and standard errors of HbO changes are shown in red for pre-THC, and black for post-THC. Error bars represent standard deviations.

Repeated-measure ANOVAs revealed a significant effect of drug treatment condition (pre- vs. post-THC) on HbO concentration in 6 out of the 20 channels (**Table 2**). After controlling for multiple comparisons using the Benjamini and Hochberg FDR procedure (Benjamini and Hochberg, 1995), three channels remained significant, all on the right dorsolateral PFC (ch 16, ch 19, and ch 20) (**Table 2** and **Figure 5**). Grand averaged waveforms of statistically significant HbO concentration changes in the aforementioned channels are shown in **Figure 6**. There was no significant effect of memory load (0-back vs. 2-back) on HbO concentration in any channel, and no significant interactions between treatment condition and memory load (all *p*s > 0.1). No correlations were found between the mean HbO concentration changes and *n*-back behavioral performance during the 2-back condition.

**Table 2:** Mean changes in oxyhemoglobin (HbO) concentration before and after dronabinol administration (pre and post-THC) measured in 20 prefrontal NIRS channels during 2-Back task condition.

Area	Channel	Pre-THC		Post-THC		Individual *p*-value	Corrected *p*-value^1^
		Mean	*SD*	Mean	*SD*		
**Left PFC**							
	1	0.0521	0.2593	0.0896	0.3020	0.422	0.50
	2	–0.0783	0.2850	0.0226	0.2976	0.286	0.45
	3	0.0442	0.1469	0.0837	0.1582	0.371	0.50
	4	–0.0547	0.2185	0.2949	0.3999	0.045^∗^	0.17
	5	–0.1246	0.1665	–0.0598	0.2723	0.452	0.50
	6	–0.2141	0.2591	0.1668	0.4471	0.031^∗^	0.17
	7	–0.2330	0.2018	–0.0446	0.4182	0.260	0.45
	8	–0.1474	0.3864	0.0149	0.3655	0.163	0.36
	11	0.0521	0.2593	0.0736	0.4299	0.023^∗^	0.17
**Middle PFC**							
	9	–0.1188	0.2054	–0.0206	0.4325	0.734	0.74
	12	–0.2699	0.2419	–0.0318	0.3609	0.150	0.24
**Right PFC**							
	10	–0.0416	0.3151	0.1024	0.3979	0.402	0.44
	13	0.3465	0.1978	–0.0075	0.4371	0.126	0.14
	14	0.3465	0.1978	0.0321	0.3079	0.076	0.09
	15	0.3465	0.1978	0.0626	0.2063	0.079	0.14
	16	0.3465	0.1978	0.2394	0.2272	0.005^∗^	0.04^∗^
	17	0.3465	0.1978	0.0084	0.2279	0.288	0.42
	18	0.3465	0.1978	0.1275	0.3577	0.428	0.58
	19	0.3465	0.1978	0.4136	0.2732	0.013^∗^	0.04^∗^
	20	0.3465	0.1978	0.1508	0.0915	0.007^∗^	0.04^∗^

*^1^*p*-value was corrected for multiple comparisons using the Benjamini and Hochberg FDR procedure. ^∗^*p* < 0.05.*

Finally, as an alternative analysis, we subtracted the 0-back from the 2-back portion of the task for both pre and post- THC administration [PRE (2-back – 0-back) vs. POST (2-back – 0-back)], in order to investigate the relative change in activation due to WM load between pre and post-THC administration. There were no differences between pre and post-THC administration by WM load (all *p*s > 0.1), further indicating that THC did not affect the relative change in activation associated with an increased WM load.

**FIGURE 7:**
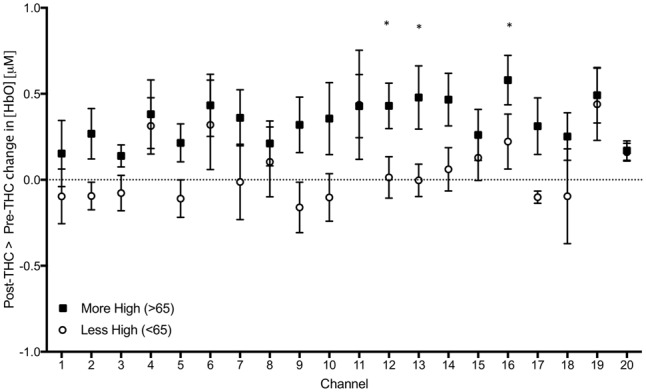
Subtraction of mean changes in HbO in each channel during 2-Back working memory task from post to pre-THC administration in those who reported feeling more (black) and less (white) high. Error bars are standard error of the mean. Channels with significant differences between groups (*p* < 0.05) are marked with an asterisk.

As an exploratory, *post hoc* analysis, we examined whether participants who reported a greater subjective ‘high’ had greater signal changes. We performed a median split between participants; group 1 included participants who felt less “high” (<65 out of possible 100; *n* = 6), and group 2 included participants who felt more “high” (>65; *n* = 7), and conducted two-sample *t*-tests between groups. We found a significant difference in three channels; Ch 12 (*t* = 2.30, *p* = 0.04), Ch 13 (*t* = 2.21, *p* = 0.04), and Ch 17 (*t* = 2.27, *p* = 0.04), indicating that those with greater subjective intoxication had greater HbO signal change detected with functional NIRS (**Figure 7**). Correlations between subjective high and signal change from pre to post-THC were not significant (all *p*s > 0.05).

## Discussion

To our knowledge, this is the first report of fNIRS to examine the effect of THC administration on prefrontal hemodynamic changes during a WM task. In this preliminary study, we observed a significant increase in HbO concentration after THC administration in several channels on the right PFC during 2-back WM condition. Though behaviorally we did not observe a performance decrement (likely due to the small sample size of 12 participants), increase in HbO may indicate that maintaining task performance was more difficult after THC administration.

An increase in HbO is consistent with the majority of brain imaging studies of smoked or orally administered cannabis. Previous studies have found that many effects of THC, including subjective levels of intoxication (Mathew and Wilson, 1992, 1993; Mathew et al., 1992), dissociative experiences, measures of depersonalization (Mathew and Wilson, 1993), measures of confusion (Mathew and Wilson, 1993), and changes in heart rate (Mathew and Wilson, 1992), correlate with increased global CBF after smoking marijuana cigarettes. Additionally, increased global CBF has been correlated with plasma THC levels (Mathew and Wilson, 1992). Task-based fMRI studies have also reported an increase in rCBF while participants were performing a cognitive task after acute THC (Borgwardt et al., 2008; Fusar-Poli et al., 2009).

Though the current study only assessed PFC changes, previous studies have indicated regional variation in the effects of THC throughout the brain. O’Leary et al. (2002), using PET, reported increased rCBF in the orbital and mesial frontal lobes (as well as in the insula, temporal poles, anterior cingulate, and cerebellum), but did not find mean global (whole-brain) CBF changes after THC administration. In fact, rCBF decreased after THC administration in auditory regions, visual cortex, parietal lobe, posterior frontal lobe, and thalamus, and rCBF was unchanged in the nucleus accumbens, basal ganglia, and hippocampus (O’Leary et al., 2002). The authors suggest that regional differences provide evidence that increased blood flow in the PFC specifically may in part underlie changes in executive functioning and/or mood that occur after THC exposure. O’Leary et al. (2007) also demonstrated that PFC increases were independent of the cognitive task being performed, indicating that changes in blood flow were likely the direct effect of the neurophysiological response of the brain and neurovascular system to THC, rather than task-related. Resting-state studies also support this conclusion (Mathew et al., 1992, 1997). In the current study, we generally observed increased signal during both the 0-back and 2-back portions of the task, though significance was only shown during the 2-back portion. This indicates that THC likely increases blood flow to the PFC irrespective of task difficulty, but this increased blood flow may be more pronounced when participants expend more effort.

Though we observed increased signal throughout the PFC, only channels in the right lateral orbitofrontal cortex (OFC) showed significantly increased blood flow after corrections for multiple comparisons. Other studies have demonstrated that cannabis users show altered structure (Cheetham et al., 2012) and function (Bolla et al., 2005) of the OFC specifically, raising the hypothesis that this region may by particularly susceptible to the effects of THC. The OFC is part of a neural network that underlies many processes in addiction and reward (Grant et al., 1996; Wang et al., 1999; Bartzokis et al., 2000; Bonson et al., 2002), and previous studies have found that alterations in this region have been associated with faulty decision-making (Bechara et al., 1994; Bolla et al., 2005). Though speculative, it is possible that chronic changes in OFC function are related to direct effects of drugs on this region during periods of acute intoxication. Other recreational drugs, such as methamphetamine (Volkow et al., 2001), cocaine (Volkow et al., 2005), and alcohol (Volkow et al., 2007), are also associated with altered function in the OFC, suggesting that several classes of psychoactive drugs may have mechanisms that contribute to deficits in prefrontal function that are commonly seen in addiction (Goldstein and Volkow, 2011).

There are many limitations to this study, and results should be interpreted cautiously. First, it is difficult to precisely measure vascular effects of THC on cerebral blood vessels (Ohlsson et al., 1980; Hollister et al., 1981), as THC does have a dilative effect on conjunctival and muscle blood vessels (Weiss et al., 1972; Ohlsson et al., 1980; Hollister et al., 1981). Previous fNIRS studies report that signal measured through fNIRS may in part arise from systemic changes that are not directly related to brain activity (Tachtsidis et al., 2008; Kirilina et al., 2012). However, in a PET study, smoked marijuana had no effect on forehead skin perfusion (Mathew et al., 1992), yet had effects on CBF. Furthermore, THC-induced vascular changes are likely to be global, while THC-induced changes in HbO in our study showed significant regional variations. Orbitofrontal channels showed significant changes, while medial ventral PFC generally did not. Furthermore, blood pressure did not change throughout the study, which is important to note given that blood pressure fluctuations can exert confounding effects on brain NIRS (Minati et al., 2011). However, we cannot rule out the possibility of drug-induced vascular changes contributing to the HbO response. In future studies, we plan to investigate the impact of systemic changes through the use of portable devices to monitor respiration, heart rate, or other physiological factors that could influence the fNIRS signal.

Second, this study was open-label, and a more rigorous exploration of the pharmacological effects of THC on cortical hemodynamics requires a double-blind placebo-controlled design. Factors such as expectation could have played a role in differences detected. Moreover, dose determination was partially based on self-report of cannabis use, and we were only able to grossly estimate the potency (THC content) of MJ used, which may influence participants’ tolerance and response to dronabinol.

Third, the current experimental design compared performance and fNIRS measurements before and after THC administration, and therefore, order and learning effects associated with *n*-back task could have influenced the results. However, this would bias the results against, rather than for, finding any differences between pre and post-THC. For separate sessions of the same task, several studies have shown greater magnitude of activation for the first session than for the second session, for both inhibitory control tasks (Langenecker and Nielson, 2003) and for language tasks (Schecklmann et al., 2008). Effects of habituation (Loubinoux et al., 2001; Fischer et al., 2003; Kiehl and Liddle, 2003) would also result in greater activation in the first than in the second task. It can therefore be expected that the HbO amplitude of the second measurement would be reduced, whereas in our study, we found that THC administration resulted in increased HbO amplitude in the second session. Furthermore, performance would be expected to improve with practice from the first to the second session, though we saw performance worsen somewhat (though not statistically significantly) after THC administration. A further methodological limitation is the use of different block lengths for the 2-back and 0-back conditions (30s vs. 20s). We chose to design the study in this way to control for the number of target trials, and because we were primarily interested in comparing each condition from post-THC to pre-THC, but the direct comparison between the 2-back and 0-back conditions may be affected by the different block lengths.

This study is also limited by the small sample size, which prevents exploration of effects such as dose, patterns of cannabis use, or interactions with factors such as gender or age. Our exploratory analysis of subjective drug response, which utilized a median split to show that participants who reported greater “high” from dronabinol exhibited greater signal increases from THC, may be especially prone to a false positive, and should be replicated in larger sample sizes with more robust statistical techniques. Given that this study represents the first report of fNIRS detecting changes in hemodynamic activity during cannabis intoxication, we chose to included this data in order to generate hypothesis that can be tested further in placebo-controlled studies with greater numbers of participants. Finally, though for ethical reasons, we could not administer dronabinol to cannabis-naïve individuals, the absence of a control group limits this study’s generalizability to non-regular cannabis users.

## Conclusion

This study demonstrates that THC administration causes increased HbO concentration changes in the PFC, supporting rCBF findings in the PET literature. Though preliminary, the current study provides the first experimental demonstration that fNIRS may be used to investigate changes in hemodynamic activity during cannabis intoxication. This easily applied, economical, and well-tolerated method presents significant advantages over PET, which is expensive, labor-intensive, and exposes participants to radiation. Because fNIRS can be used repeatedly throughout the day on a single participant, future studies can use this technique to investigate the time-course of acute intoxication, and recovery from intoxication, which could greatly improve our understanding of the brain’s response to an intoxicating substance.

## Author Contributions

Conceived and designed the experiments: JMG, AEE, and HOK. Performed the experiments: HOK and MR. Oversaw clinical aspects: GP and AEE. Analyzed the data: HOK and JMG. Wrote the paper: JMG, HOK, and MR. All authors have approved the final article.

## Conflict of Interest Statement

AEE received research grant funding and/or study supplies to her institution from Forum Pharmaceuticals, GSK, and Pfizer, and has performed consulting work for Reckitt Benckiser and Pfizer. JMG and AEE hold a provisional patent on use of fNIRS to determine impairment due to cannabis intoxication. The other authors declare that the research was conducted in the absence of any commercial or financial relationships that could be construed as a potential conflict of interest.
